# Alcohol consumption and the risk of liver disease: a nationwide, population-based study

**DOI:** 10.3389/fmed.2023.1290266

**Published:** 2023-11-28

**Authors:** Sang Yi Moon, Minkook Son, Yeo Wool Kang, Myeongseok Koh, Jong Yoon Lee, Yang Hyun Baek

**Affiliations:** ^1^Division of Gastroenterology, Department of Internal Medicine, Dong-A University College of Medicine, Busan, Republic of Korea; ^2^Department of Physiology, Dong-A University College of Medicine, Busan, Republic of Korea

**Keywords:** alcohol, alcoholic liver disease, chronic hepatitis, liver cirrhosis, nation-wide population-based study

## Abstract

**Introduction:**

Although most patients with alcohol-related liver disease (ALD) have a history of prolonged and heavy drinking, there is no clear threshold defining the level of alcohol consumption that leads to ALD. We aimed to evaluate the correlation between average alcohol consumption and the risk of liver disease and to determine the threshold for clinically significant alcohol consumption.

**Materials and methods:**

Using the Korean National Health Insurance database, we identified participants who underwent a health-screening program in 2010 and 2011 and retrospectively analyzed their data until 2019. To diagnose and categorize the extracted participants, we used the International Classification of Diseases version 10 and Fatty Liver Index. The primary outcome was to determine the incidence of newly diagnosed liver-related diseases during the observation period and compare the incidence of liver-related diseases among non-drinkers and drinkers based on the amount of alcohol consumption.

**Results:**

A total of 53,006 patients were enrolled and followed-up for a median of 8.4 years, during which 1,509 cases of liver-related diseases occurred. The participants were divided into five groups: no alcohol consumption (*n* = 31,359), 1st quartile (*n* = 5,242), 2nd quartile (*n* = 5,704), 3rd quartile (*n* = 5,337), and 4th quartile (*n* = 5,364). The corresponding number of glasses of alcohol consumed per week for each quantile (Q1, Q2, Q3, and Q4) was labeled 2.5 ± 1.1 standard units (1 standard unit = 8 g alcohol), 5.4 ± 1.9 standard units, 11.5 ± 3.3 standard units, and 27.9 ± 18.2 standard units, respectively. Compared with non-drinkers, the risk of liver-related disease was found to be higher in Q1 drinkers (adjusted hazard ratio [aHR], 1.09; 95% CI, 0.90–1.33), Q2 drinkers (aHR, 1. 10; 95% CI, 0.91–1.32), Q3 drinkers (aHR, 1.33; 95% CI, 1.11–1.59), and Q4 drinkers (aHR, 1.47; 95% CI, 1.24–1.75).

**Conclusion:**

We report that our study has shown that drinking more than 11.5 ± 3.3 standard units/week (92 ± 26.4 g/week) significantly increases the risk of developing liver-related diseases. Therefore, as a preventive measure to reduce the risk of developing liver disease, alcohol consumption should be limited beyond traditionally recommended levels.

## 1 Introduction

Alcohol has been used extensively in various cultures for centuries, playing a central role in religious practices and nutrition and enriching the overall quality and enjoyment of life (1). The effects of alcohol on health are a combination of positive and negative effects, and research has shown that moderate drinking can have beneficial physical, mental, and social effects (2). However, excessive alcohol consumption is among the major contributors to several diseases (3), including several types of cancer (4), diabetes mellitus (DM) (5), hypertension (6), atrial fibrillation (7), pancreatitis (8), dementia (9), and depression (10). It frequently affects the liver and results in alcohol-related liver disease (ALD) (11, 12). ALD presents with a broad variety of clinical manifestations and pathological characteristics such as isolated steatosis, progressive steatohepatitis with fibrosis accumulation, cirrhosis, and related complications (13). Although several factors such as sex, age, race, nutrition, genetics, and pre-existing liver disease contribute to the development and progression of ALD, a history of excessive alcohol consumption remains an indispensable criterion for ALD diagnosis (14).

Globally, the incidence of chronic liver disease (CLD) and cirrhosis has increased by 13% since 2000, with an estimated rate of 20.7 per 100,000 individuals in 2015 (15). A study investigating the causes of cirrhosis between 1990 and 2017 revealed that the incidence of cirrhosis caused by viral hepatitis, including hepatitis B and C, increased modestly to 29 and 28% of cases, respectively, which is likely attributable to the effectiveness of highly targeted vaccines and antiviral therapies (15). In contrast, cirrhosis associated with ALD and nonalcoholic fatty liver disease (NAFLD), both of which lack a standard treatment for disease progression, exhibited a more pronounced trend, demonstrating significant increases of 78 and 125%, respectively (15). Moreover, the 2016 Global Status Report on Alcohol and Health indicated that nearly half of the deaths related to liver disease, that is, 588,100 of 1,254,000 cases, resulted from alcohol consumption (16). Based on these data, ALD remains a major contributor to CLD, emphasizing the importance of defining a threshold for heavy drinking as a crucial diagnostic measure for ALD (17). Nevertheless, different organizations and academic associations have developed inconsistent definitions of “heavy drinking” that may lead to the development of ALD, and “moderate drinking,” which is considered non-harmful to health (18).

This study identified liver disease diagnoses caused by alcohol consumption based on average alcohol consumption in a large, representative sample of South Koreans. Subsequently, we established a benchmark for clinically significant alcohol consumption by comparing non-drinkers and drinkers.

## 2 Materials and methods

### 2.1 Data source

The Korean government’s mandatory health insurance system covers nearly 97% of the South Korean population, with the remaining 3% covered by the Medical Aid Program. In South Korea, the biennial health examination program aims to provide comprehensive coverage for all employees regardless of age, and for individuals aged 40 and older. Based on the health screening data, the National Health Insurance Service has developed and provided various national health information databases (19, 20). These include the National Health Insurance Service-National Health Screening Cohort (NHIS-HealS) database, which was created by selecting 10% of the approximately 5 million individuals who participated in the health screening program in 2002 and 2003, resulting in a cohort of 514,866 individuals (21). The NHIS-HealS database was used for this study, and contains comprehensive data on medical claims, including prescription records, procedures, surgeries, and insurance premium payments and covers both inpatient and outpatient services. Based on the health screening data, in addition to physical measurements and laboratory tests, a self-report questionnaire on lifestyle was administered.

### 2.2 Ethics statement

This study protocol was exempted from review by the Institutional Review Board of the Dong-A University College of Medicine because of the retrospective design of the study, and the researchers accessed only de-identified open clinical data for analytical purposes (DAUHIRB-EXP-23-106).

### 2.3 Study population

**Figure 1:**
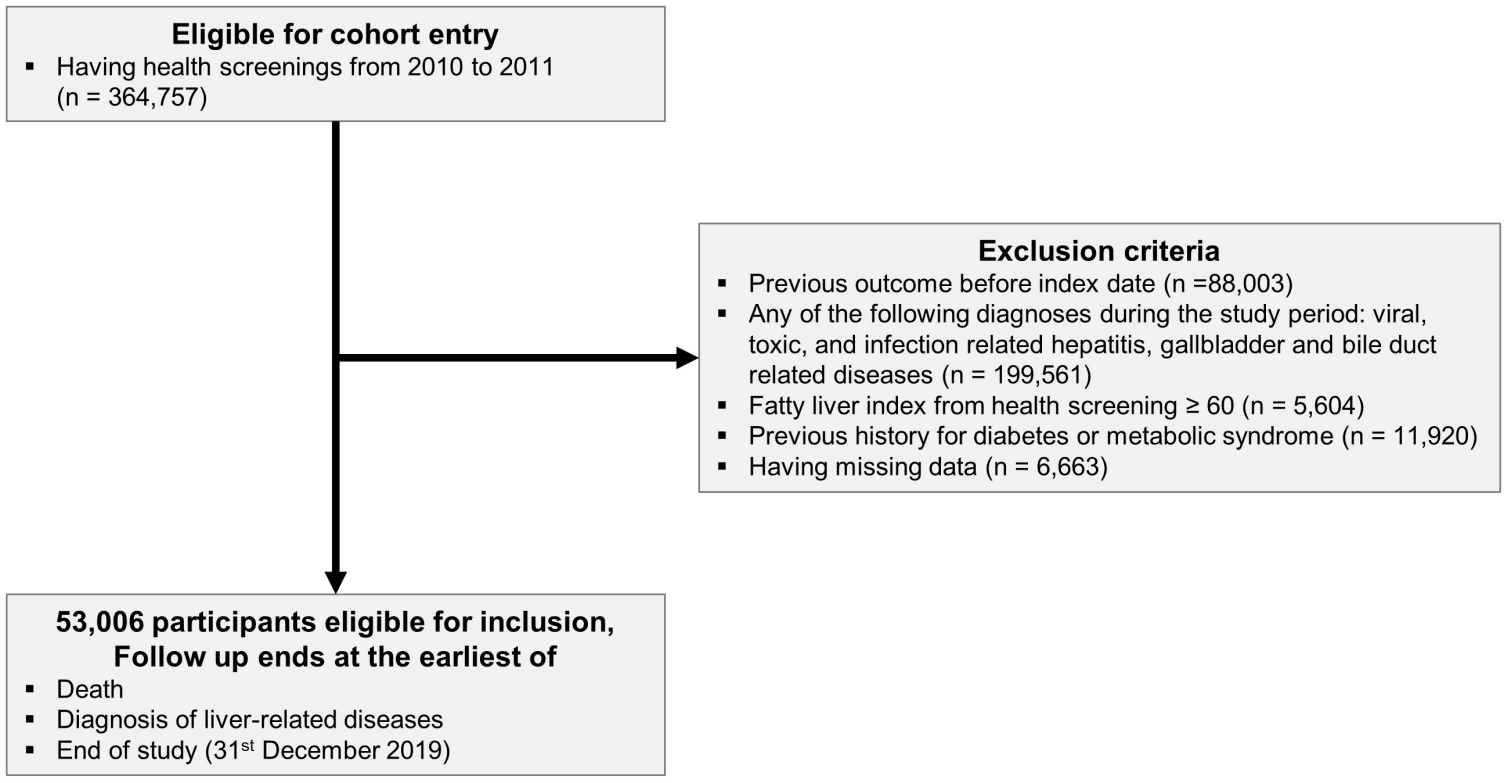
The flow of study population.

We identified participants who took part in a health-screening program in 2010 and 2011 (n = 364,757). We used the International Classification of Disease version 10 (ICD-10) and Fatty Liver Index (FLI) to diagnose and categorize the extracted participants. We excluded previous diagnoses of liver-related diseases (n = 88,003), diagnoses of any cause of liver disease during the observation period (viral, toxic, infection-related hepatitis, gallbladder, and bile duct-related disease) (n = 199,561), and missing data (n = 6,663) (Supplementary Table S1). Using health screening data, we calculated the FLI to diagnose NAFLD (22). Participants with an FLI ≥ 60 were excluded from the study due to their high likelihood of having NAFLD (n = 5,604). Participants with a history of DM or metabolic syndrome (n = 11, 920) were excluded. Finally, 53,006 participants were included in the final analysis (Figure 1).

### 2.4 Exposure variable: alcohol consumption

The health-screening questionnaire provided data on the frequency (number of days per week) and intensity (number of drinks per occasion) of alcohol consumption, allowing the determination of an individual’s drinking behavior. To ensure consistency, a standard drink was defined as a specific cup size for each type of alcohol including beer, soju (traditional Korean alcohol), and whiskey. Although the cups varied in volume, they were designed to contain an equivalent amount of alcohol (8 g) (23). The average alcohol consumption (glasses per week) was calculated as the product of frequency and intensity. The participants were categorized into two groups: non-drinkers and drinkers. Within the drinker group, four quantiles (Q1, Q2, Q3, and Q4) were established based on the average alcohol consumption for each sex.

### 2.5 Outcome variable: liver-related disease and follow-up

Liver-related diseases were identified using the following ICD-10 codes: ALD (ICD-10: K70), liver failure (ICD-10: K72), chronic hepatitis (ICD-10: K73), and cirrhosis (ICD-10: K740, K741, K742, and K746). Participants were followed up from the index date (date of health screening) until the diagnosis of liver-related disease, death, or December 31, 2019, whichever came first.

### 2.6 Covariates

Demographic information, including age, sex, and comorbidities, including hypertension and dyslipidemia, were assessed based on diagnoses from the medical claims and health-screening databases (Supplementary Table S1). The Charlson comorbidity index (CCI) was calculated based on comorbidities (24). Body mass index (BMI) was calculated as body weight in kilograms divided by the square of the height in meters (kg/m2). The laboratory results included aspartate aminotransferase, alanine aminotransferase, r-glutamyl transpeptidase, hemoglobin level, and glomerular filtration rate. The self-report questionnaire consisted of information on current smoking and regular exercise status. The income level was categorized into four quantiles, and residence was categorized into urban and rural.

### 2.7 Statistical analyses

Categorical variables are reported as counts with percentages, while continuous variables are presented as means with standard deviations.

Using the Kaplan–Meier curve, we assessed the incidence of liver-related diseases over time in non-drinkers and drinker groups (categorized into four quantiles) with average alcohol consumption. The association between average alcohol consumption and liver-related diseases was expressed as a hazard ratio (HR) with a 95% confidence interval (CI) using the Cox proportional hazards model. To compare the incidence of liver-related diseases according to alcohol consumption, we categorized drinkers into four quantiles, with non-drinkers as the reference group, and compared the HRs. The proportional hazard assumption was evaluated using the Schoenfeld residuals test with the logarithm of the cumulative hazard function based on Kaplan–Meier estimates. There was no disturbance under the assumption of a proportional hazard risk over time. Multi-variable adjusted models included covariates including age, sex, income level, residence, hypertension, dyslipidemia, CCI, BMI, aspartate aminotransferase, alanine aminotransferase, γ-glutamyl transpeptidase, hemoglobin level, glomerular filtration rate, smoking, and regular exercise status.

The nonlinear dose–response relationship between average alcohol consumption and the incidence of liver-related disease was represented by a restricted cubic spline. We used four knots at the 5th, 35th, 65th, and 95th percentiles according to the average alcohol consumption (25, 26). The mean value of the average alcohol consumption was selected as the reference value for the restricted cubic spline.

Subgroup analyses were performed according to sex and age (<65 and ≥ 65 years) in male participants.

All statistical analyses were conducted using SAS version 9.4 (SAS Institute Inc., Cary, NC, USA) and R 3.6.0 (R Foundation for Statistical Computing, Vienna, Austria). Statistical significance was defined as *p* < 0.05.

## 3 Results

### 3.1 Baseline characteristics of study population

A total of 53,006 participants were included in this study and categorized into five groups: no alcohol consumption (n = 31,359), 1st quartile (n = 5,242), 2nd quartile (n = 5,704), 3rd quartile (n = 5,337), and 4th quartile (n = 5,364). The non-drinkers was 36.9% males and 63.1% females, and the corresponding number of glasses of alcohol consumed per week for each quantile (Q1, Q2, Q3, and Q4) was labeled 1.8 ± 1.1 standard units (1 standard unit = 8 g alcohol), 5.1 ± 2.1 standard units, 10.4 ± 3.9 standard units, and 24.6 ± 14.6 standard units, respectively. At the 2011–2012 health screening, there were significant differences (*p* < 0.001) among the five groups for all characteristics (Table 1). The baseline characteristics of each sex are shown in Supplementary Tables S2 and S3.

**Table 1:** Baseline characteristics of study population.

All participants (*n* = 53,006)	Average alcohol consumption					*p*-value
	No alcohol (*N* = 31,359)	1st quartile, Q1 (*N* = 5,242)	2nd quartile, Q2 (*n* = 5,704)	3rd quartile, Q3 (*n* = 5,337)	4th quartile, Q4 (*n* = 5,364)	
Demographics						
Age (years)	58.6 ± 8.6	57.0 ± 7.6	55.5 ± 6.7	55.5 ± 6.8	55.5 ± 7.0	< 0.001
Sex (%)						< 0.001
Male	11,574 (36.9%)	4,661 (88.9%)	4,347 (76.2%)	4,820 (90.3%)	4,416 (82.3%)	
Female	19,785 (63.1%)	581 (11.1%)	1,357 (23.8%)	517 (9.7%)	948 (17.7%)	
Income level (%)						< 0.001
1st quartile	4,820 (15.4%)	563 (10.7%)	656 (11.5%)	498 (9.3%)	605 (11.3%)	
2nd quartile	6,846 (21.8%)	820 (15.6%)	994 (17.4%)	930 (17.4%)	1,048 (19.5%)	
3rd quartile	8,696 (27.7%)	1,350 (25.8%)	1,504 (26.4%)	1,404 (26.3%)	1,474 (27.5%)	
4th quartile	10,997 (35.1%)	2,509 (47.9%)	2,550 (44.7%)	2,505 (46.9%)	2,237 (41.7%)	
Residence (%)						< 0.001
Urban	20,008 (63.8%)	3,753 (71.6%)	4,103 (71.9%)	3,794 (71.1%)	3,587 (66.9%)	
Rural	11,351 (36.2%)	1,489 (28.4%)	1,601 (28.1%)	1,543 (28.9%)	1,777 (33.1%)	
Underlying disease						
Hypertension (%)	9,034 (28.8%)	1,418 (27.1%)	1,415 (24.8%)	1,400 (26.2%)	1,546 (28.8%)	< 0.001
Dyslipidemia (%)	8,050 (25.7%)	1,154 (22.0%)	1,140 (20.0%)	983 (18.4%)	915 (17.1%)	< 0.001
Charlson comorbidity index						< 0.001
0	20,254 (64.6%)	3,737 (71.3%)	4,187 (73.4%)	4,042 (75.7%)	4,033 (75.2%)	
1	7,585 (24.2%)	1,113 (21.2%)	1,188 (20.8%)	997 (18.7%)	1,052 (19.6%)	
2	2,470 (7.9%)	299 (5.7%)	239 (4.2%)	238 (4.5%)	227 (4.2%)	
≥3	1,050 (3.3%)	93 (1.8%)	90 (1.6%)	60 (1.1%)	52 (1.0%)	
Health screening						
Body mass index (kg/m^2^)	22.9 ± 2.6	23.0 ± 2.3	23.1 ± 2.3	23.1 ± 2.3	23.1 ± 2.3	< 0.001
Waist circumference (cm)	77.7 ± 7.3	80.4 ± 6.5	79.9 ± 6.8	80.8 ± 6.4	80.7 ± 6.4	< 0.001
Systolic blood pressure (mmHg)	121.0 ± 14.7	121.8 ± 13.8	121.7 ± 13.9	123.3 ± 13.7	124.4 ± 14.3	< 0.001
Diastolic blood pressure (mmHg)	74.9 ± 9.6	76.2 ± 9.5	76.4 ± 9.4	77.5 ± 9.3	78.1 ± 9.5	< 0.001
Fasting blood glucose (mg/dL)	92.1 ± 10.0	93.3 ± 10.5	93.4 ± 10.5	94.5 ± 10.6	95.0 ± 10.9	< 0.001
Total cholesterol (mg/dL)	200.2 ± 34.5	195.6 ± 32.4	197.8 ± 32.8	196.7 ± 32.2	196.9 ± 33.0	< 0.001
Triglyceride (mg/dL)	106.6 ± 53.1	108.8 ± 52.6	110.1 ± 53.8	114.7 ± 56.6	115.4 ± 57.1	< 0.001
HDL cholesterol (mg/dL)	57.0 ± 19.8	54.9 ± 18.2	56.8 ± 16.2	57.1 ± 17.3	59.3 ± 13.9	< 0.001
LDL cholesterol (mg/dL)	122.2 ± 32.5	119.3 ± 32.3	119.1 ± 32.3	117.0 ± 32.7	114.7 ± 33.1	< 0.001
Aspartate aminotransferase (U/L)	23.2 ± 7.5	23.7 ± 8.4	23.7 ± 9.4	24.3 ± 9.4	25.2 ± 11.1	< 0.001
Alanine aminotransferase (U/L)	19.7 ± 9.7	21.2 ± 10.9	20.7 ± 11.1	20.9 ± 9.3	21.0 ± 9.8	< 0.001
r-glutamyl transpeptidase (U/L)	20.9 ± 13.7	27.1 ± 16.9	28.8 ± 20.4	35.2 ± 25.2	40.2 ± 33.2	< 0.001
Hemoglobin (g/dL)	13.4 ± 1.4	14.5 ± 1.3	14.3 ± 1.4	14.6 ± 1.2	14.5 ± 1.3	< 0.001
Glomerular filtration rate (mL/min/1.73 m^2^)	80.2 ± 27.4	80.0 ± 38.8	81.3 ± 39.0	81.9 ± 40.0	82.3 ± 28.5	< 0.001
Fatty liver index	15.7 ± 12.4	20.0 ± 13.8	20.6 ± 14.3	23.8 ± 15.1	25.1 ± 15.5	< 0.001
Current smoker (%)	2,812 (9.0%)	1,167 (22.3%)	1,522 (26.7%)	1,840 (34.5%)	20,72 (38.6%)	< 0.001
Regular exercise (%)	1,332 (4.2%)	255 (4.9%)	278 (4.9%)	242 (4.5%)	259 (4.8%)	0.05
Average alcohol consumption (standard units per week)						
All	0.0 ± 0.0	2.5 ± 1.1	5.4 ± 1.9	11.5 ± 3.3	27.9 ± 18.2	< 0.001
Male	0.0 ± 0.0	2.7 ± 1.0	6.4 ± 1.1	12.3 ± 2.4	31.2 ± 17.6	< 0.001
Female	0.0 ± 0.0	1.0 ± 0.0	2.4 ± 0.5	4.3 ± 0.4	12.5 ± 1.19	< 0.001
Outcome						
Liver-related disease (%)	837 (2.7)	134 (2.6)	150 (2.6)	173 (3.2)	215 (4.0)	< 0.001
Alcohol-related liver disease (%)	131 (0.4)	34 (0.6)	49 (0.9)	78 (1.5)	100 (1.9)	< 0.001
Liver failure (%)	57 (0.2)	13 (0.2)	9 (0.2)	14 (0.3)	10 (0.2)	0.61
Chronic hepatitis (%)	613 (2.0)	80 (1.5)	87 (1.5)	76 (1.4)	102 (1.9)	0.009
Liver cirrhosis (%)	36 (0.1)	7 (0.1)	5 (0.1)	5 (0.1)	3 (0.1)	0.71

### 3.2 Incidence and risk of liver-related disease according to average alcohol consumption

**Figure 2:**
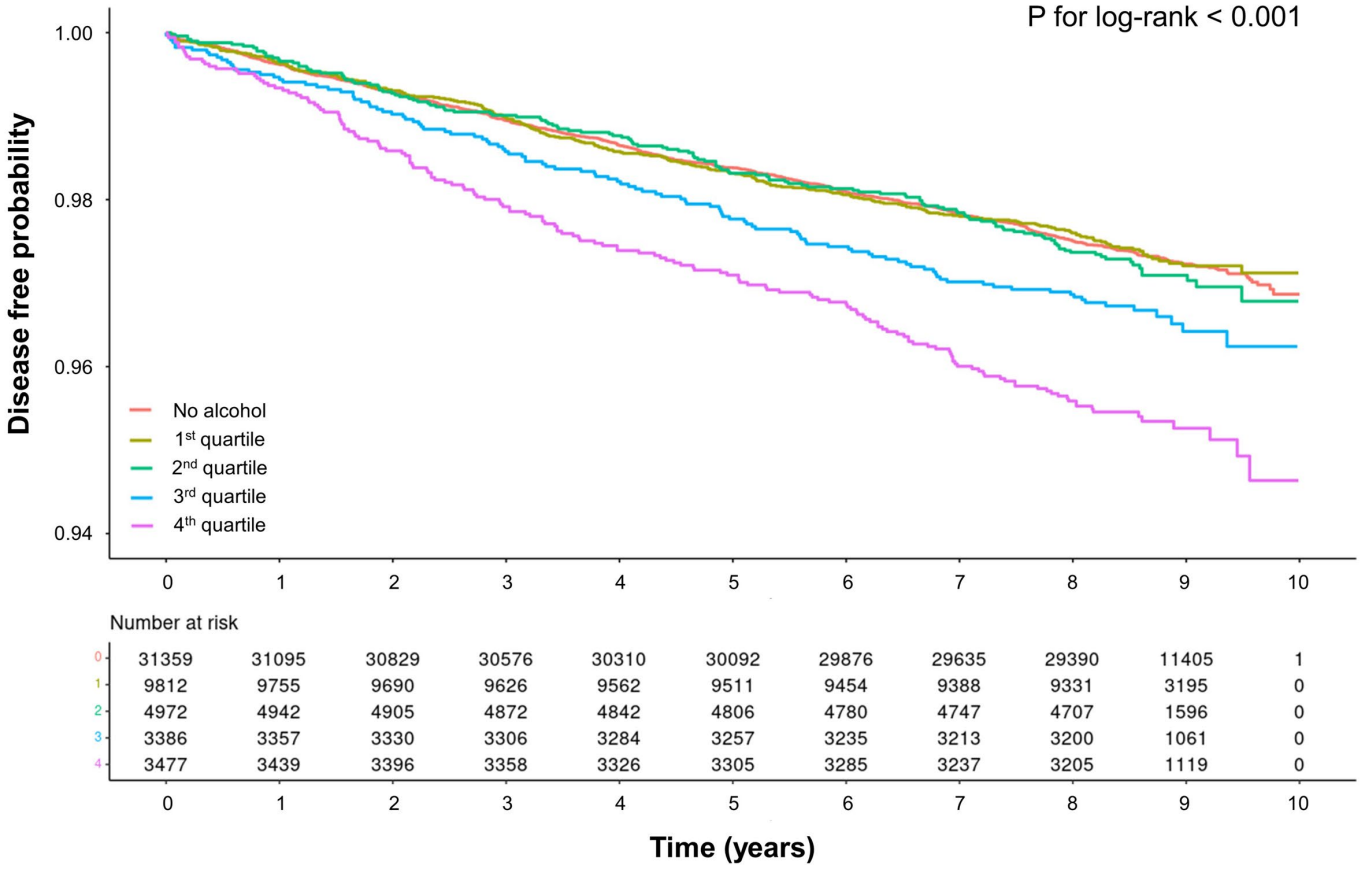
Kaplan–Meier curve for association between average alcohol consumption and liver-related diseases.

The median follow-up duration of the study population was 8.4 years, during which 1,509 cases of liver-related diseases occurred. We performed Kaplan–Meier analysis to compare the cumulative incidence rates of liver-related diseases among non-drinkers, Q1 drinkers, Q2 drinkers, Q3 drinkers, and Q4 drinkers (Figure 2). The analysis revealed a significant increase in the risk of liver-related diseases in Q3 drinkers and Q4 drinkers compared to non-drinkers (*p* < 0.001, log-rank test). This visualization highlights a significant association between average alcohol consumption and the occurrence of liver-related diseases in the Q3 and Q4 drinker groups.

In the crude analysis, the incidence rates of liver-related diseases in non-drinkers, Q1 drinkers, Q2 drinkers, Q3 drinkers, and Q4 drinkers were 3.14, 3.02, 3.09, 3.83, and 4.76 cases per 1,000 person-years, respectively (Table 2). The corresponding HR and 95% CIs for liver-related disease were 1 (reference), 0.96 (95% CI, 0.80–1.15), 0.99 (95% CI, 0.83–1.17), 1.22 (95% CI, 1.04–1.44), and 1.52 (95% CI, 1.30–1.76) in the same groups. After adjusting for variables, the HRs for liver-related disease were 1 (reference), 1.09 (95% CI, 0.90–1.33), 1.10 (95% CI, 0.91–1.32), 1.33 (95% CI, 1.11–1.59), and 1.47 (95% CI, 1.24–1.75) for non-drinkers, Q1 drinkers, Q2 drinkers, Q3 drinkers, and Q4 drinkers, respectively. When comparing them with non-drinkers as the reference group, statistically significant associations with average alcohol consumption were observed in Q3 drinkers and Q4 drinkers.

**Table 2:** Hazard ratio and 95% confidence interval for incidence of liver-related diseases according to average alcohol consumption.

	Events	Follow-up duration (person-years)	Incidence rate (per 1,000 person-years)	Hazard ratio (95% confidence intervals)			
				Crude	P-value	Adjusted*	*p*-value
All participants (*n* = 53,006)							
No	837	266,417	3.14	1.00 (reference)		1.00 (reference)	
Q1	134	44,374	3.02	0.96 (0.80–1.15)	0.68	1.09 (0.90–1.33)	0.37
Q2	150	48,474	3.09	0.99 (0.83–1.17)	0.87	1.10 (0.91–1.32)	0.32
Q3	173	45,147	3.83	1.22 (1.04–1.44)	0.02	1.33 (1.11–1.59)	0.002
Q4	215	45,189	4.76	1.52 (1.30–1.76)	< 0.001	1.47 (1.24–1.75)	< 0.001
Male participants (*n* = 29,818)							
No	277	96,583	2.87	1.00 (reference)		1.00 (reference)	
Q1	118	39,365	3.00	1.05 (0.84–1.30)	0.68	1.18 (0.95–1.46)	0.15
Q2	111	36,804	3.02	1.05 (0.85–1.31)	0.65	1.22 (0.97–1.53)	0.08
Q3	159	40,707	3.91	1.36 (1.12–1.66)	0.002	1.49 (1.22–1.83)	< 0.001
Q4	184	37,053	4.97	1.73 (1.44–2.09)	< 0.001	1.65 (1.35–2.03)	< 0.001
Female participants (*n* = 23,188)							
No	560	169,834	3.30	1.00 (reference)		1.00 (reference)	
Q1	16	5,009	3.19	0.97 (0.59–1.59)	0.90	1.03 (0.62–1.69)	0.92
Q2	39	11,670	3.34	1.02 (0.73–1.40)	0.93	1.04 (0.75–1.45)	0.80
Q3	14	4,440	3.15	0.96 (0.56–1.63)	0.87	1.04 (0.61–1.78)	0.88
Q4	31	8,136	3.81	1.16 (0.81–1.66)	0.43	1.16 (0.80–1.68)	0.45

*The model was adjusted for age, sex, income level, residence, hypertension, dyslipidemia, Charlson comorbidity index, body mass index, aspartate aminotransferase, alanine aminotransferase, r-glutamyl transpeptidase, hemoglobin level, glomerular filtration rate, smoking, and regular exercise status.

### 3.3 Nonlinear dose–response curves of average alcohol consumption and liver-related disease: Restricted cubic spline

**Figure 3:**
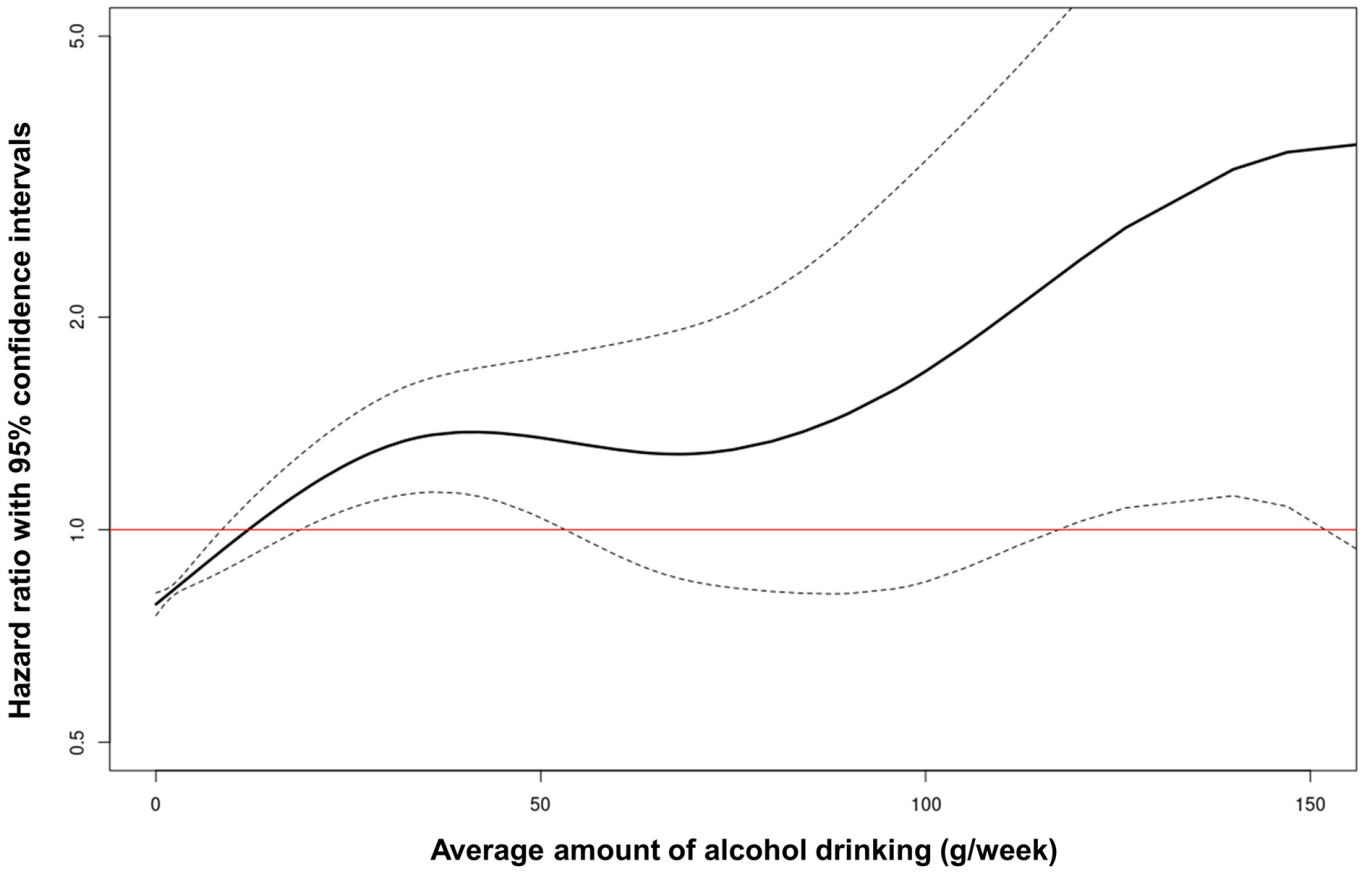
Restricted cubic spline of hazard ratio with 95% confidence intervals for liver-related diseases according to average alcohol consumption.

The non-linear relationship between average alcohol consumption and liver-related diseases was evaluated using a restricted cubic spline with a multivariable adjusted model to depict the trend of HR with respect to average alcohol consumption. The restricted cubic spline curve showed that the risk for liver-related diseases increased with the total amount of alcohol consumed (Figure 3).

### 3.4 Subgroup analyses

The results of subgroup analyses by sex are shown in Table 2, and the results of subgroup analyses by age (<65 and ≥ 65 years) in male participants are shown in Supplementary Table S4. The subgroup analysis for male participants was consistent with the main analysis; however, this was not observed for female participants. In addition, the subgroup analysis of male participants by age (<65 and ≥ 65 years) showed a higher HR in the older participants (≥65 years).

## 4 Discussion

This large national cohort study found that an elevated risk of liver-related diseases was associated with average alcohol consumption. A nearly proportional association was found between the average amount of alcohol consumed and the risk of liver-related diseases. In comparison to non-drinkers, the consumption of 11.5 ± 3.3 standard units/week (92 ± 26.4 g/week) is significantly associated with increased prevalence of liver-related disease. The resulting values in our study were lower than the thresholds for significant alcohol consumption suggested in previous clinical practice guidelines (14, 27, 28).

The association between the average amount of alcohol consumption and the diagnosis of liver disease was the result of careful consideration of the study population and outcomes. To exclude causes of CLD other than alcohol consumption, the study population was defined using the ICD-10 codes. Viral hepatitis (ICD-10: B15, B16, B17, B18, and B19), toxic hepatitis (ICD-10: K71), biliary cirrhosis (ICD-10: K743, K744, and K745), other inflammatory liver diseases (ICD-10: K75), other liver diseases (ICD-10: K76), and gallbladder or biliary tract diseases (ICD-10: K80, K81, K82, and K83) were excluded. Moreover, to enhance the accuracy of NAFLD identification, we used health screening data and calculated the FLI, which allowed us to identify cases that might have been missed by relying solely on diagnostic codes (22). Participants with an FLI ≥ 60 were excluded from the study due to their increased likelihood of having NAFLD. Additionally, to ensure a more homogeneous study population, participants with a history of DM or metabolic syndrome that could potentially influence NAFLD development were excluded from the analysis (27, 29, 30). Concerning the outcome, Pang JX et al. (31) found a low positive predictive value of 54% (95% CI, 0.47–0.60) when validating ALD using only ICD codes. For this reason, in addition to ALD (ICD-10: K70), we have included the ‘liver-related disease’ of liver failure (ICD-10: K72), chronic hepatitis (ICD-10: K73), and cirrhosis (ICD-10: K740, K741, K742, and K746) as outcomes, given that these can be classified as CLD (32, 33). This process prioritized alcohol as the cause of liver disease and broadened the scope of ALD to encompass ‘liver-related disease,’ thus corroborating the relationship between average alcohol consumption and liver disease.

Is there a defined standard for the safe consumption of alcohol termed ‘moderate drinking’ (18)? The public health policy generally aims to establish safe levels of alcohol consumption, often referred to as ‘moderate drinking,’ although specific criteria may vary among organizations (12). The National Institute on Alcohol Abuse and Alcoholism in the United States defines clinically significant alcohol consumption as consuming more than 14 standard drinks per week for men and more than seven for women (where one standard drink equals 14 g of alcohol), whereas the European Association for the Study of the Liver and Korean Association for the Study of the Liver indicate that clinically significant alcohol consumption exceeds 210 g per week for men and 140 g per week for women (18). Research on the effects of moderate drinking on disease outcomes is a continually evolving area, and some previous studies have indicated a potential reduction in the risk of cardiovascular diseases (34, 35). However, concerns about the potential harm associated with even moderate drinking have been raised in several recent studies. A study utilizing the United Kingdom Biobank found that even small amounts of alcohol are associated with an increased risk of cardiovascular disease, and that the cardioprotective effect of moderate drinking is largely due to lifestyle factors (36). According to a Finnish population survey with cross-sectional analysis, consuming alcohol other than wine within the range of 0–9 grams was associated with a two-fold increase in advanced liver disease in patients diagnosed with hepatic steatosis (37). In a biopsy-confirmed study of NAFLD with concurrent DM, the group consuming 66–140 grams of alcohol per week had a significantly higher incidence of advanced fibrosis than the group consuming less than 66 g per week (50.0–60.0% vs. 3.3–21.6%, *p* < 0.05) (38). In addition, alcohol is a known carcinogen, and consumption of any amount is not considered safe owing to the risk of cancer (4). Regular drinking of amounts greater than small (≥1 drink/day for women and ≥ 2 drink/day for men) was significantly associated with increased risk of liver cancer (odds ratio:1.418, 95% CI, 1.192 to 1.687; *p* < 0.001) (39). Additionally, a meta-analysis exploring the association between alcohol use and suicide demonstrated a higher risk of suicide with a risk ratio of 1.65 (95% CI, 1.33 to 2.05; *p* < 0.001) (40).

The varying outcomes for disease associated with alcohol consumption can be attributed to a number of factors, such as sex, coexisting medical conditions, variety of alcoholic beverages, binge drinking, and genetics; however, the study design also affects the results. The effects of alcohol consumption often require a significant amount of time to become clear, which frequently results in the use of observational studies with cross-sectional designs to define moderate drinking. However, this approach can cause problems, such as selection bias and reverse causality (41). Additionally, one study indicated that recall bias can underestimate alcohol consumption by up to 40–50%, a factor that may have affected investigations of the correlation between actual alcohol consumption and disease (42). Despite these limitations, many researchers are developing evidence-based definitions for moderate and heavy drinking. Our study has shown that drinking more than 11.5 ± 3.3 standard units/week (92 ± 26.4 g/week) significantly increases the risk of developing liver-related diseases. Therefore, it is necessary to reduce the limit of clinically significant alcohol intake that can potentially lead to liver diseases.

When analyzing the risk based on sex, we did not find any significant association between liver-related diseases and alcohol consumption in female participants. It is possible that the lack of an observed association was due to the sample size limitations. Of the 23,188 female participants during the study period, 19,785 (85.3%) were classified as non-drinkers. Only 948 participants in the female Q4 group consumed alcohol at a level significantly associated with liver-related diseases in the overall group, making it challenging to demonstrate statistical significance.

Our study has several limitations. First, as this was a cross-sectional study, changes in drinking behavior could not be confirmed, and the non-drinking group may have included high-risk participants who opted out because of underlying health issues. This may have led to light drinkers being healthier. Second, the self-reported data on drinking frequency and daily alcohol consumption may have been inaccurate. Third, the study lacked data on genetic polymorphisms of alcoholic enzymes, which are important factors in the development of alcoholic liver disease. Fourth, despite the exclusion of diagnoses affecting CLD through ICD codes, we were unable to collect clear information regarding viral markers or other factors that could have influenced the study participants. Despite the aforementioned limitations, this study used data from a large sample of over 53,000 people collected over a period of eight years. The study utilized measures such as fasting laboratory findings, anthropometric parameters, lifestyle behaviors, and systematic data collection on diagnosis from a standardized database, all in an effort to minimize the potential bias associated with self-reporting.

In conclusion, we report that our study has shown that drinking more than 11.5 ± 3.3 standard units/week (92 ± 26.4 g/week) significantly increases the risk of developing liver-related diseases. The increasing prevalence of CLD associated with ALD is a growing global concern. However, the lack of established pharmaceutical interventions or treatment strategies to counteract and reverse ALD progression of ALD is still a significant hurdle. Therefore, as a preventive measure to reduce the risk of developing liver disease, alcohol consumption should be limited beyond traditionally recommended levels. Following the basic medical principle of “first, do no harm” with regard to alcohol consumption may be beneficial.

## Data availability statement

The datasets presented in this study can be found in online repositories. The names of the repository/repositories and accession number(s) can be found in the article/Supplementary material.

## Ethics statement

The studies involving humans were approved by Dong-A University Hospital IRB. The studies were conducted in accordance with the local legislation and institutional requirements. The ethics committee/institutional review board waived the requirement of written informed consent for participation from the participants or the participants’ legal guardians/next of kin because a nationwide, population-based study.

## Author contributions

SM: Conceptualization, Data curation, Formal analysis, Investigation, Methodology, Visualization, Writing – original draft. MS: Conceptualization, Data curation, Formal analysis, Methodology, Software, Visualization, Writing – original draft. YK: Validation, Writing – review & editing. MK: Validation, Writing – review & editing. JL: Validation, Writing – review & editing. YB: Conceptualization, Funding acquisition, Project administration, Resources, Writing – review & editing.
