# Supplementary material for: Alcohol consumption and the risk of liver disease: a nationwide, population-based study
Source: Front Med (Lausanne). 2023 Nov 28;10:1290266. doi: 10.3389/fmed.2023.1290266 (PMC10713769; doi:10.3389/fmed.2023.1290266)
Supplement: Supplementary file 1 [file Table_1.DOCX]

**Supplementary materials**

**Supplementary Table S1. Definitions for clinical variables.**

| **Covariates** | **ICD-10 codes + Claim codes** | **Health screening** |
| --- | --- | --- |
| Hypertension | I10-I11 + Prescription of anti-hypertensive drugs ≥ 1 | Systolic blood pressure ≥ 140 mmHg or Diastolic blood pressure ≥ 90 mmHg |
| Diabetes | E10-E14 + Prescription of anti-diabetic drugs ≥ 1 | Fasting blood glucose ≥ 126 mg/dL |
| Dyslipidemia | E78 + Prescription of lipid-lowering drugs ≥ 1 | Total cholesterol 240 mg/dL or  LDL cholesterol 190 mg/dL or  Triglyceride 500 mg/dL or  HDL cholesterol < 40 mg/dL |
| **Outcome** | **ICD-10 codes** | **Claim codes** |
| Liver-related diseases | K70, K72, K73,  K740, K741, K742, K746 | ≥1 admission or ≥2 outpatients visits |
| **Exclusion criteria** | **ICD-10 codes** | **Claim codes** |
| Viral hepatitis | B15, B16, B17, B18, B19 | ≥ 1 admission or outpatient visit |
| Toxic hepatitis | K71 | ≥ 1 admission or outpatient visit |
| Biliary cirrhosis | K743, K744, K745 | ≥ 1 admission or outpatient visit |
| Other inflammatory liver diseases | K75 | ≥ 1 admission or outpatient visit |
| Other liver disease | K76 | ≥ 1 admission or outpatient visit |
| Gallbladder or biliary tract disease | K80, K81, K82, K83 | ≥ 1 admission or outpatient visit |

**Supplementary Table S2. Baseline characteristics of male population.**

| **All subjects**  **(n = 29,818)** | **Average alcohol consumption** | | | | | **P-value** |
| --- | --- | --- | --- | --- | --- | --- |
|  | **No alcohol**  **(n=11,574)** | **1^st^ quartile, Q1**  **(n=4,661)** | **2^nd^ quartile, Q2**  **(n=4,347)** | **3^rd^ quartile, Q3**  **(n=4,820)** | **4^th^ quartile, Q4**  **(n=4,416)** |  |
| **Demographics** |  |  |  |  |  |  |
| Age (years) | 59.0 ± 8.8 | 57.1 ± 7.6 | 55.9 ± 6.9 | 55.7 ± 6.8 | 55.7 ± 7.1 | < 0.001 |
| Income level (%) |  |  |  |  |  | < 0.001 |
| 1^st^ quartile | 1,400 (12.1%) | 446 (9.6%) | 411 (9.5%) | 398 (8.3%) | 431 (9.8%) |  |
| 2^nd^ quartile | 2,234 (19.3%) | 705 (15.1%) | 650 (15.0%) | 787 (16.3%) | 772 (17.5%) |  |
| 3^rd^ quartile | 3,307 (28.6%) | 1,205 (25.9%) | 1,159 (26.7%) | 1,265 (26.2%) | 1,199 (27.2%) |  |
| 4^th^ quartile | 4,633 (40.0%) | 2,305 (49.5%) | 2,127 (48.9%) | 2,370 (49.2%) | 2,014 (45.6%) |  |
| Residence (%) |  |  |  |  |  | < 0.001 |
| Urban | 7,563 (65.3%) | 3,330 (71.4%) | 3,092 (71.1%) | 3,404 (70.6%) | 2,917 (66.1%) |  |
| Rural | 4,011 (34.7%) | 1,331 (28.6%) | 1,255 (28.9%) | 1,416 (29.4%) | 1,499 (33.9%) |  |
| **Underlying disease** |  |  |  |  |  |  |
| Hypertension (%) | 3,411 (29.5%) | 1,284 (27.5%) | 1,115 (25.6%) | 1,291 (26.8%) | 1,329 (30.1%) | < 0.001 |
| Dyslipidemia (%) | 2,847 (24.6%) | 1,024 (22.0%) | 835 (19.2%) | 891 (18.5%) | 722 (16.3%) | < 0.001 |
| Charlson comorbidity index |  |  |  |  |  | < 0.001 |
| 0 | 7,792 (67.3%) | 3,358 (72.0%) | 3,283 (75.5%) | 3,668 (76.1%) | 3,372 (76.4%) |  |
| 1 | 2,592 (22.4%) | 968 (20.8%) | 850 (19.6%) | 886 (18.4%) | 832 (18.8%) |  |
| 2 | 789 (6.8%) | 254 (5.4%) | 159 (3.7%) | 213 (4.4%) | 174 (3.9%) |  |
| ≥3 | 401 (3.5%) | 81 (1.7%) | 55 (1.3%) | 53 (1.1%) | 38 (0.9%) |  |
| **Health screening** |  |  |  |  |  |  |
| Body mass index (kg/m^2^) | 22.9 ± 2.4 | 23.0 ± 2.3 | 23.1 ± 2.3 | 23.1 ± 2.3 | 23.1 ± 2.3 | < 0.001 |
| Systolic blood pressure (mmHg) | 122.3 ±14.3 | 122.2 ±13.8 | 122.8 ±13.6 | 123.8 ±13.5 | 125.1 ±14.2 | < 0.001 |
| Diastolic blood pressure (mmHg) | 76.1 ± 9.4 | 76.6 ± 9.4 | 77.0 ± 9.3 | 77.8 ± 9.2 | 78.7 ± 9.5 | < 0.001 |
| Fasting blood glucose (mg/dL) | 93.3 ±10.6 | 93.5 ±10.6 | 93.9 ±10.6 | 94.8 ±10.6 | 95.5 ±11.0 | < 0.001 |
| Total cholesterol (mg/dL) | 193.1 ±33.2 | 194.4 ±32.2 | 195.6 ±32.2 | 196.0 ±32.0 | 195.1 ±32.5 | < 0.001 |
| Triglyceride (mg/dL) | 112.9 ±57.0 | 110.1 ±53.0 | 114.6 ±54.3 | 116.9 ±57.1 | 119.0 ±58.6 | < 0.001 |
| HDL cholesterol (mg/dL) | 52.4 ±17.8 | 54.0 ±18.5 | 54.7 ±14.7 | 56.4 ±17.4 | 58.0 ±13.4 | < 0.001 |
| LDL cholesterol (mg/dL) | 118.5 ±31.2 | 118.8 ±32.6 | 118.1 ±32.6 | 116.8 ±32.8 | 113.6 ±33.2 | < 0.001 |
| Aspartate aminotransferase (U/L) | 23.5 ± 7.3 | 23.8 ± 7.9 | 23.9 ± 7.8 | 24.5 ± 9.4 | 25.7 ±11.8 | < 0.001 |
| Alanine aminotransferase (U/L) | 21.7 ±10.0 | 21.5 ±10.2 | 21.4 ± 9.3 | 21.2 ± 9.5 | 21.7 ±10.0 | 0.05 |
| r-glutamyl transpeptidase (U/L) | 25.5 ±15.8 | 28.1 ±17.2 | 31.7 ±20.6 | 36.8 ±25.5 | 43.8 ±34.5 | < 0.001 |
| Hemoglobin (g/dL) | 14.5 ± 1.2 | 14.7 ± 1.1 | 14.8 ± 1.1 | 14.8 ± 1.1 | 14.8 ± 1.1 | < 0.001 |
| Glomerular filtration rate (mL/min/1.73 m^2^) | 79.6 ±31.8 | 79.7 ±38.9 | 81.3 ±43.0 | 82.0 ±41.6 | 82.3 ±29.5 | < 0.001 |
| Current smoker (%) | 2,658 (23.0%) | 1,161 (24.9%) | 1,505 (34.6%) | 1,828 (37.9%) | 2,002 (45.3%) | < 0.001 |
| Regular exercise (%) | 609 (5.3%) | 234 (5.0%) | 227 (5.2%) | 218 (4.5%) | 213 (4.8%) | 0.33 |
| Average alcohol consumption (standard units per week) | 0.0 ± 0.0 | 2.7 ± 1.0 | 6.4 ± 1.1 | 12.3 ± 2.4 | 31.2 ±17.6 | < 0.001 |

**Supplementary Table S3. Baseline characteristics of female population.**

| **All subjects**  **(n = 23,188)** | **Average alcohol consumption** | | | | | **P-value** |
| --- | --- | --- | --- | --- | --- | --- |
|  | **No alcohol**  **(n=19,785)** | **1^st^ quartile, Q1**  **(n=581)** | **2^nd^ quartile, Q2**  **(n=1,357)** | **3^rd^ quartile, Q3**  **(n=517)** | **4^th^ quartile, Q4**  **(n=948)** |  |
| **Demographics** |  |  |  |  |  |  |
| Age (years) | 58.3 ± 8.5 | 56.3 ± 8.1 | 54.2 ± 6.1 | 54.3 ± 6.4 | 54.8 ± 6.6 | < 0.001 |
| Income level (%) |  |  |  |  |  | < 0.001 |
| 1^st^ quartile | 3420 (17.3%) | 117 (20.1%) | 245 (18.1%) | 100 (19.3%) | 174 (18.4%) |  |
| 2^nd^ quartile | 4612 (23.3%) | 115 (19.8%) | 344 (25.4%) | 143 (27.7%) | 276 (29.1%) |  |
| 3^rd^ quartile | 5389 (27.2%) | 145 (25.0%) | 345 (25.4%) | 139 (26.9%) | 275 (29.0%) |  |
| 4^th^ quartile | 6364 (32.2%) | 204 (35.1%) | 423 (31.2%) | 135 (26.1%) | 223 (23.5%) |  |
| Residence (%) |  |  |  |  |  | < 0.001 |
| Urban | 12445 (62.9%) | 423 (72.8%) | 1011 (74.5%) | 390 (75.4%) | 670 (70.7%) |  |
| Rural | 7340 (37.1%) | 158 (27.2%) | 346 (25.5%) | 127 (24.6%) | 278 (29.3%) |  |
| **Underlying disease** |  |  |  |  |  |  |
| Hypertension (%) | 5623 (28.4%) | 134 (23.1%) | 300 (22.1%) | 109 (21.1%) | 217 (22.9%) | < 0.001 |
| Dyslipidemia (%) | 5203 (26.3%) | 130 (22.4%) | 305 (22.5%) | 92 (17.8%) | 193 (20.4%) | < 0.001 |
| Charlson comorbidity index |  |  |  |  |  | < 0.001 |
| 0 | 12462 (63.0%) | 379 (65.2%) | 904 (66.6%) | 374 (72.3%) | 661 (69.7%) |  |
| 1 | 4993 (25.2%) | 145 (25.0%) | 338 (24.9%) | 111 (21.5%) | 220 (23.2%) |  |
| 2 | 1681 (8.5%) | 45 (7.7%) | 80 (5.9%) | 25 (4.8%) | 53 (5.6%) |  |
| ≥3 | 649 (3.3%) | 12 (2.1%) | 35 (2.6%) | 7 (1.4%) | 14 (1.5%) |  |
| **Health screening** |  |  |  |  |  |  |
| Body mass index (kg/m^2^) | 22.9 ± 2.6 | 22.7 ± 2.4 | 22.9 ± 2.4 | 23.0 ± 2.3 | 23.1 ± 2.5 | 0.02 |
| Systolic blood pressure (mmHg) | 120.2 ±14.9 | 118.5 ±13.8 | 118.5 ±14.5 | 119.2 ±14.3 | 121.0 ±14.3 | < 0.001 |
| Diastolic blood pressure (mmHg) | 74.2 ± 9.6 | 73.1 ± 9.3 | 74.3 ± 9.7 | 74.6 ± 9.2 | 75.3 ± 9.4 | 0.001 |
| Fasting blood glucose (mg/dL) | 91.4 ± 9.6 | 91.5 ± 9.6 | 91.8 ±10.0 | 91.8 ± 9.5 | 92.7 ± 9.9 | < 0.001 |
| Total cholesterol (mg/dL) | 204.3 ±34.5 | 204.7 ±32.5 | 204.7 ±33.9 | 203.2 ±33.6 | 205.3 ±33.9 | 0.83 |
| Triglyceride (mg/dL) | 102.9 ±50.3 | 98.9 ±48.2 | 95.9 ±49.3 | 93.9 ±47.2 | 98.8 ±46.4 | < 0.001 |
| HDL cholesterol (mg/dL) | 59.7 ±20.4 | 61.4 ±13.5 | 63.4 ±19.0 | 64.5 ±14.4 | 65.3 ±14.8 | < 0.001 |
| LDL cholesterol (mg/dL) | 124.4 ±33.0 | 123.4 ±29.4 | 122.3 ±31.3 | 119.7 ±31.7 | 120.1 ±32.0 | < 0.001 |
| Aspartate aminotransferase (U/L) | 23.0 ± 7.5 | 22.8 ±11.8 | 23.0 ±13.1 | 23.0 ± 8.8 | 22.9 ± 6.1 | 0.94 |
| Alanine aminotransferase (U/L) | 18.6 ± 9.4 | 18.4 ±15.0 | 18.4 ±15.3 | 17.8 ± 7.3 | 17.9 ± 7.7 | 0.08 |
| r-glutamyl transpeptidase (U/L) | 18.2 ±11.6 | 19.3 ±11.4 | 19.6 ±17.1 | 19.9 ±16.0 | 23.5 ±19.3 | < 0.001 |
| Hemoglobin (g/dL) | 12.8 ± 1.1 | 12.8 ± 1.2 | 12.9 ± 1.1 | 12.9 ± 1.0 | 13.0 ± 1.0 | < 0.001 |
| Glomerular filtration rate (mL/min/1.73 m^2^) | 80.5 ±24.4 | 82.4 ±37.8 | 81.2 ±21.3 | 80.8 ±20.5 | 82.3 ±23.1 | 0.08 |
| Current smoker (%) | 154 (0.8%) | 6 (1.0%) | 17 (1.3%) | 12 (2.3%) | 70 (7.4%) | < 0.001 |
| Regular exercise (%) | 723 (3.7%) | 21 (3.6%) | 51 (3.8%) | 24 (4.6%) | 46 (4.9%) | 0.30 |
| Average alcohol consumption (standard units per week) | 0.0 ± 0.0 | 1.0 ± 0.0 | 2.4 ± 0.5 | 4.3 ± 0.4 | 12.5 ±11.9 | < 0.001 |

**Supplementary Table S4. Subgroup analyses according to age in male subjects.**

|  | **Events** | **Follow-up duration (person-years)** | **Incidence rate (per 1,000 person-years)** | **Hazard ratio (95% confidence intervals)** | | | |
| --- | --- | --- | --- | --- | --- | --- | --- |
|  |  |  |  | **Crude** | **P-value** | **Adjusted*** | **P-value** |
| **Age < 65 (n = 24,956)** |  |  |  |  |  |  |  |
| No | 182 | 75374 | 2.41 | 1.00 (reference) |  | 1.00 (reference) |  |
| Q1 | 91 | 33735 | 2.70 | 1.12 (0.87-1.44) | 0.39 | 1.14 (0.88-1.47) | 0.31 |
| Q2 | 89 | 33176 | 2.68 | 1.11 (0.86-1.43) | 0.42 | 1.12 (0.87-1.45) | 0.38 |
| Q3 | 133 | 36713 | 3.62 | 1.50 (1.20-1.88) | < 0.001 | 1.43 (1.13-1.81) | 0.003 |
| Q4 | 150 | 33203 | 4.52 | 1.87 (1.51-2.32) | < 0.001 | 1.56 (1.24-1.97) | < 0.001 |
| **Age ≥ 65 (n = 4,862)** |  |  |  |  |  |  |  |
| No | 95 | 21209 | 4.48 | 1.00 (reference) |  | 1.00 (reference) |  |
| Q1 | 27 | 5630 | 4.80 | 1.07 (0.70-1.65) | 0.75 | 1.22 (0.79-1.88) | 0.37 |
| Q2 | 22 | 3628 | 6.06 | 1.36 (0.85-2.15) | 0.20 | 1.61 (1.00-2.58) | 0.05 |
| Q3 | 26 | 3994 | 6.51 | 1.46 (0.94-2.25) | 0.09 | 1.49 (0.95-2.33) | 0.09 |
| Q4 | 34 | 3850 | 8.83 | 1.97 (1.33-2.91) | < 0.001 | 1.82 (1.18-2.82) | 0.007 |

*The model was adjusted for age, income level, residence, hypertension, dyslipidemia, Charlson comorbidity index, body mass index, aspartate aminotransferase, alanine aminotransferase, r-glutamyl transpeptidase, hemoglobin level, glomerular filtration rate, smoking, and regular exercise status.
